# Idiopathic Mixed Cryoglobulinemia: A Diagnostic Challenge

**DOI:** 10.7759/cureus.70069

**Published:** 2024-09-24

**Authors:** Hinal Rathi, Tumpa Patra, Indira Poojary, Prutha R Pathak, Heather Haley

**Affiliations:** 1 Internal Medicine, University of Alabama Heersink School of Medicine Huntsville Regional Campus, Huntsville, USA; 2 Internal Medicine, Crestwood Medical Center, Huntsville, USA; 3 Internal Medicine, North Alabama Medical Center, Florence, USA; 4 Nephrology, Huntsville Renal Clinic, Huntsville, USA

**Keywords:** hepatitis-c, idiopathic, idiopathic cryoglobulinemia, mixed cryoglobulinemia, polyclonal cryoglobulinemia

## Abstract

Mixed cryoglobulinemia (MC) is commonly associated with chronic hepatitis C infection. Symptoms usually present as a clinical triad of purpuric rash, arthralgia, and generalized weakness. There have been several case reports establishing the relationship between hepatitis C and MC. Here, we report a case of a 22-year-old female presenting with bilateral lower extremity and facial swelling with no history of hepatitis C developing idiopathic mixed cryoglobulinemia. She was treated with intravenous steroids, rituximab, and plasmapheresis, resulting in improvement with outpatient nephrology and oncology follow-ups.

## Introduction

Cryoglobulinemia occurs when serum immunoglobulins precipitate at cold temperatures (less than 37 °C). It is characterized by a clinical triad of purpuric lesions (75%), arthralgia (44%), and generalized weakness [1,2]. The condition is estimated to be five per 100,000 persons in Europe and North America [3]. There are three types of cryoglobulinemia: Type I, II, and III. Around 90% of cases are Type II and III, which are mixed cryoglobulinemia (MC), with the latter having polyclonal IgG and IgM [1-3]. Cryoglobulinemia is associated with hepatitis C virus, autoimmune diseases, and B-cell lymphomas. The pathogenesis entails the deposition of immune complexes in small- and medium-sized vessels, triggering complement activation and causing damage to vessel walls [3,4]. Definitive diagnosis is established through measurement of serum cryo-immunoglobulins or cryocrit. A kidney biopsy can be done in cases of cryoglobulinemia causing glomerulonephritis. Treatment involves avoidance of cold exposures, glucocorticoids, and immunosuppression [4]. In extreme cases, it can cause vasculitis, which requires therapeutic plasma exchange or plasmapheresis. Additionally, in such patients, immunizations for COVID-19, influenza, varicella-zoster, and pneumococcus should be administered since this condition decreases the stability of the immune system [5].

## Case presentation

We report a case of a 22-year-old female with a past medical history significant for prior pregnancy complicated with a gunshot wound to the abdomen with acute uterine injury and fetal demise at age 19. Three years later, the patient was able to successfully conceive. She was referred to nephrology at 25 weeks gestation due to proteinuria with a urine protein to creatinine ratio (UPCR) of approximately 3 grams. Subsequently, the patient had low complement 4 of 6 mg/dl (normal: 15-45 mg/dl). Her renal function was normal with a serum creatinine of 0.6 mg/dL. Further workup included HIV, antinuclear antibodies (ANA) with reflex, antineutrophil cytoplasmic antibody (C-ANCA) titers, and anti-glomerular basement membrane (anti-GBM), all of which were negative. Given the fact that her renal function was normal and she was 25 weeks gestation, having traumatically lost her first child, following lengthy discussions with obstetrics-gynecology, it was felt that we should follow her expectantly and consider empiric corticosteroids if proteinuria worsened. Fortunately, her proteinuria stabilized to a UPCR of approximately 1.5 grams at 37 weeks. Also of note, she developed right hydronephrosis during pregnancy, was evaluated by urology, and followed with no intervention required. The patient successfully delivered a full-term baby girl via scheduled C-section. The patient was lost to follow-up immediately postpartum.

Four months following delivery, the patient presented to the emergency department complaining of bilateral lower extremity and facial swelling that had progressed over the last week. She denied any shortness of breath, chest pain, fevers, or chills. The patient also denied any use of tobacco, alcohol, or illicit substances. Vital signs on admission were a temperature of 36.8 °C, heart rate of 64 beats per minute, respiratory rate of 18 breaths per minute, blood pressure of 152/88 mmHg, and oxygen saturation of 99% on room air. Significant labs are mentioned in Table 1.

**Table 1:** Remarkable labs BNP: brain natriuretic peptide, Hgb: hemoglobin, Hct: hematocrit, UPCR: urine protein to creatinine ratio

Lab name	Patient value	Reference range
Protein	4.5 g/dL	6.0-8.3 g/dL
Albumin	2.2 g/dL	3.4-5.4 g/dL
Protein gap	2.3 g/dL	<4.0 g/dL
Pro-BNP	659 pg/mL	100-400 pg/mL
Complement 3	79 mg/dL	75-175 mg/dL
Complement 4	6 mg/dL	15-45 mg/dL
Hgb	9.5 g/dL	11.6-15 g/dL
Hct	29.6%	36-48%
Ferritin	12 ng/mL	13-150 ng/mL
Iron saturation	28%	15-45%
Vitamin B12	240 pg/mL	160-950 pg/mL
Creatinine	1.0 mg/dL	0.7-1.3 mg/dL
UPCR	11 grams	<30 mg

**Figure 1:**
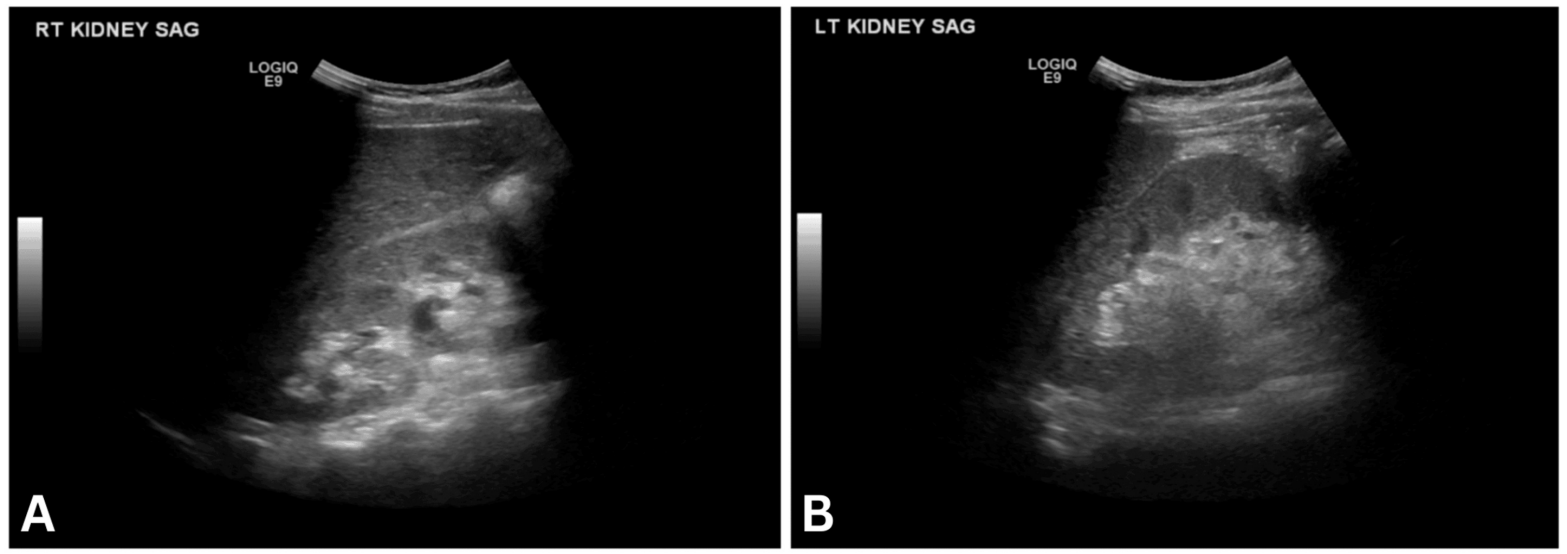
Renal ultrasound image of the right and left kidneys (A) A renal ultrasound image of the right kidney showing increased echogenicity indicative of medical renal disease with no hydronephrosis. (B) A renal ultrasound image of the left kidney showing increased echogenicity indicative of medical renal disease with no hydronephrosis.

Diagnostic workup included renal ultrasound (Figure 1), only showing relatively increased echogenicity of the renal parenchyma suggesting medical renal disease and very mild fullness of the right renal collecting system with no concerns for frank hydronephrosis. In order to rule out heart failure as a cause of bilateral lower extremity edema, she also underwent a transthoracic echocardiogram with an ejection fraction of 55-60% with no wall motion abnormalities, no significant valvular abnormalities, and normal diastolic function. Doppler of bilateral lower extremities was negative for deep vein thrombosis.

Nephrology was consulted and nifedipine 60 mg for blood pressure control, along with albumin and Lasix for anasarca related to nephrotic syndrome, was initiated. Additional workup included thyroid stimulating hormone, HIV, hepatitis panel, ANA with reflex, C- and P-ANCA, anti-GBM antibody, myeloperoxidase antibody, proteinase 3 antibody, serum and urine immunofixation, serum kappa/lambda light chain ratio, cryoglobulin level at day 1 and day 7, and anti-Ro and anti-La antibodies, all of which were negative. Rheumatoid factor was elevated at 371, but anti-cyclic citrullinated peptide (anti-CCP) antibodies were negative.

**Figure 2:**
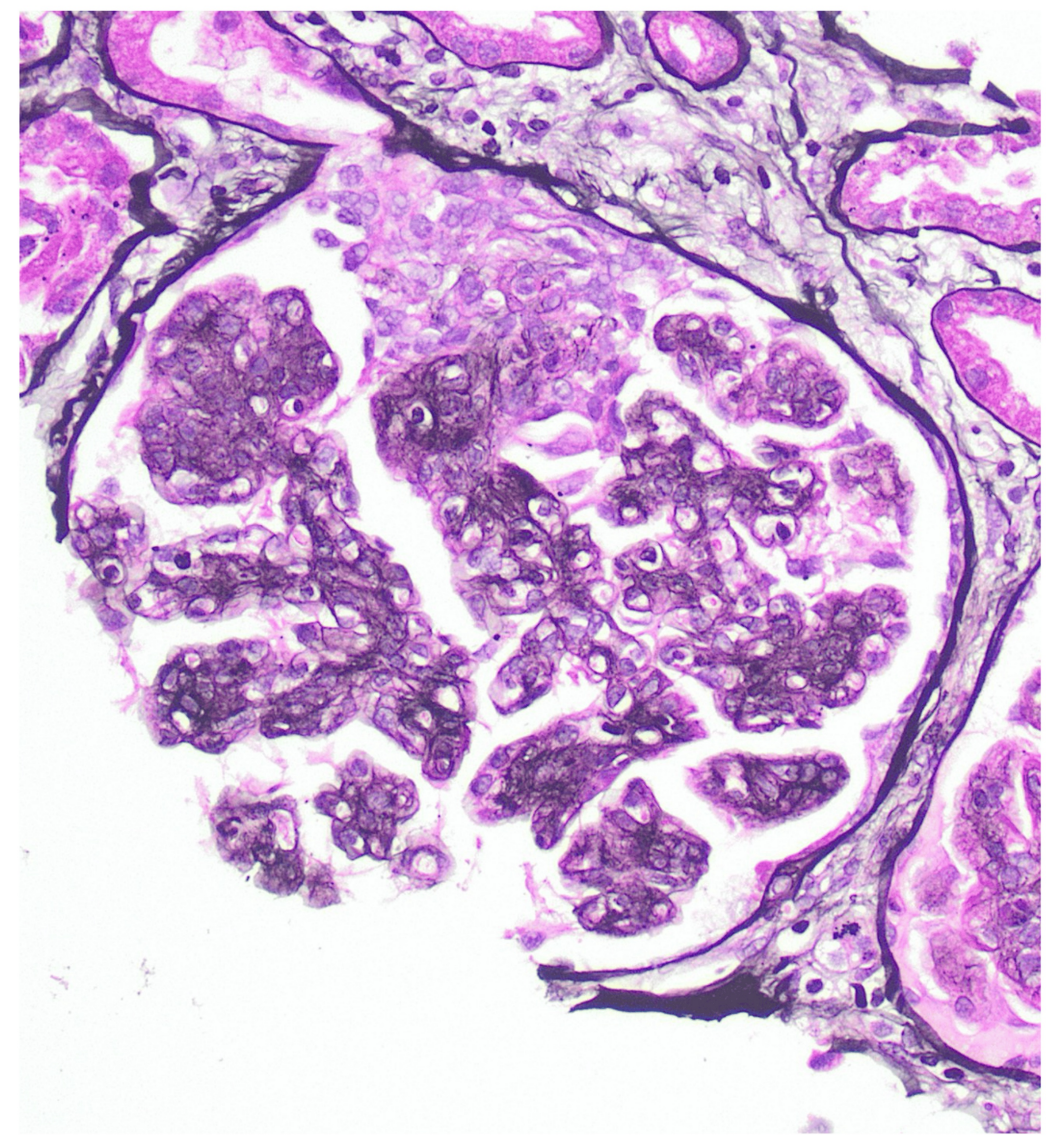
Glomerulus with fibrocellular crescent with adhesion (Jones stain, 400X) Courtesy of Dr. Paisit Paueksakon and Dr. Tiffany L. Alley at the Vanderbilt University Department of Pathology, Microbiology, and Immunology

**Figure 3:**
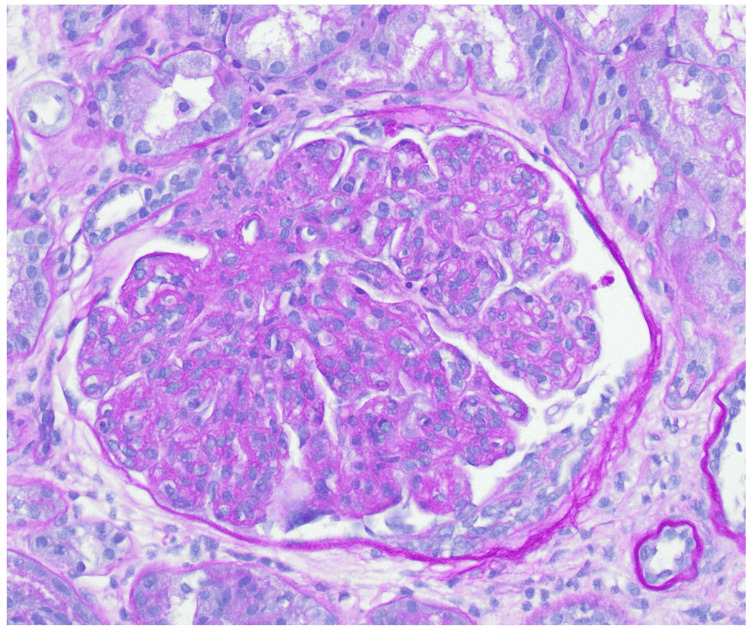
Glomerulus with cellular crescent (PAS stain, 400X) PAS: periodic acid-Schiff Courtesy of Dr. Paisit Paueksakon and Dr. Tiffany L. Alley at the Vanderbilt University Department of Pathology, Microbiology, and Immunology

**Figure 4:**
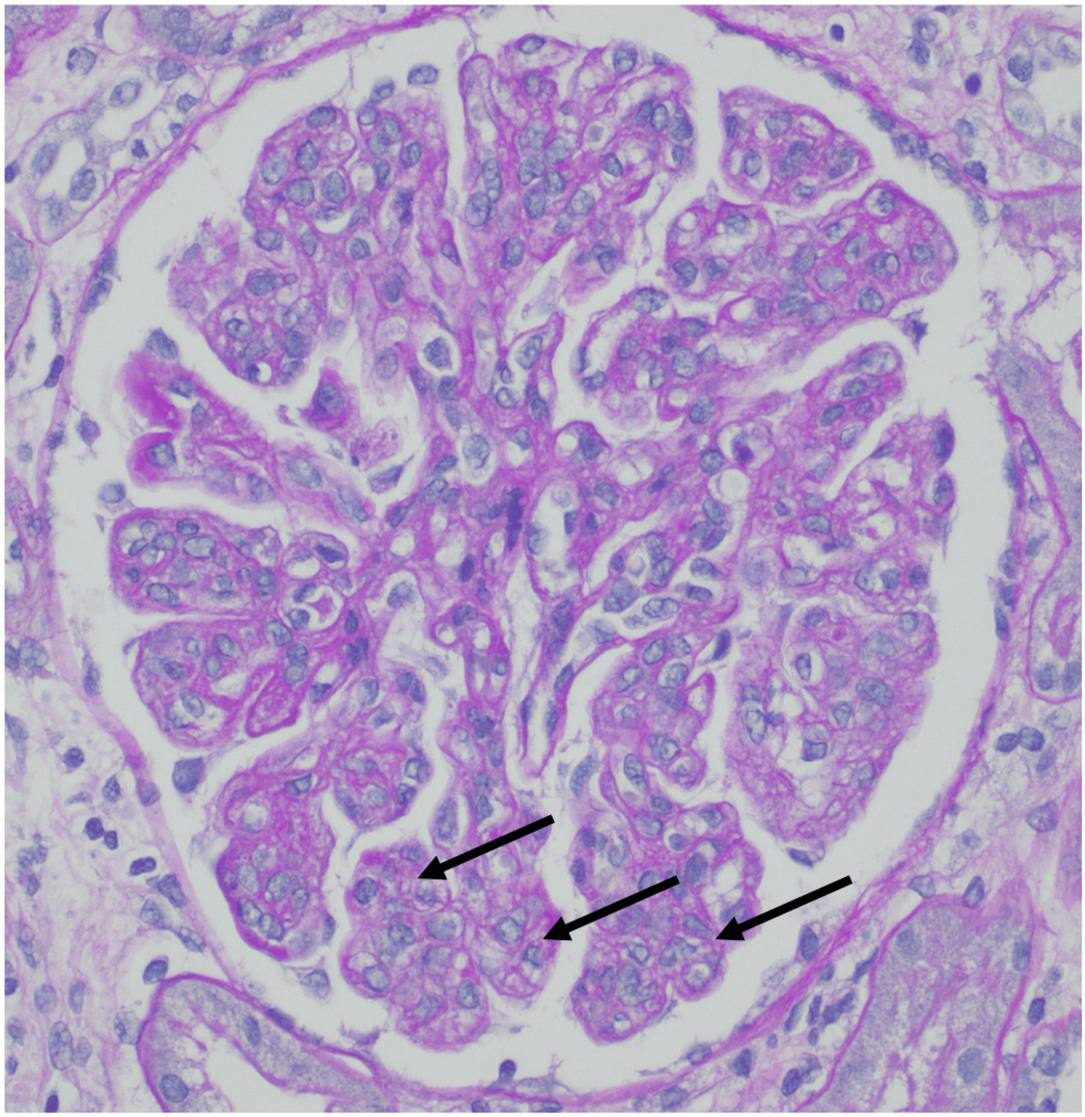
Glomerulus with endocapillary hypercellularity (PAS stain, 400X) PAS: periodic acid-Schiff Courtesy of Dr. Paisit Paueksakon and Dr. Tiffany L. Alley at the Vanderbilt University Department of Pathology, Microbiology, and Immunology

**Figure 5:**
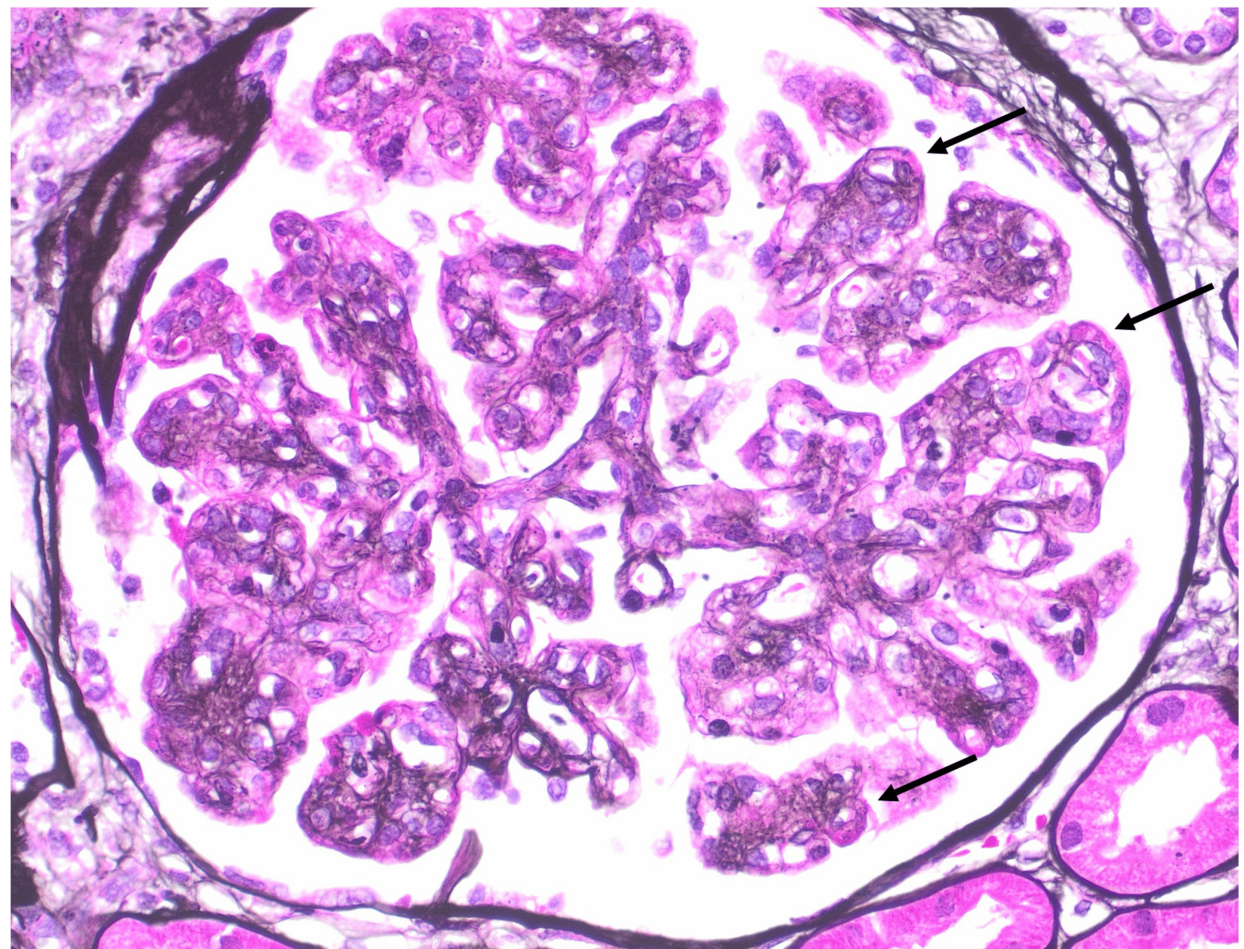
Glomerulus with double contours and eosinophilic deposits (Jones stain, 400X) Courtesy of Dr. Paisit Paueksakon and Dr. Tiffany L. Alley at the Vanderbilt University Department of Pathology, Microbiology, and Immunology

**Figure 6:**
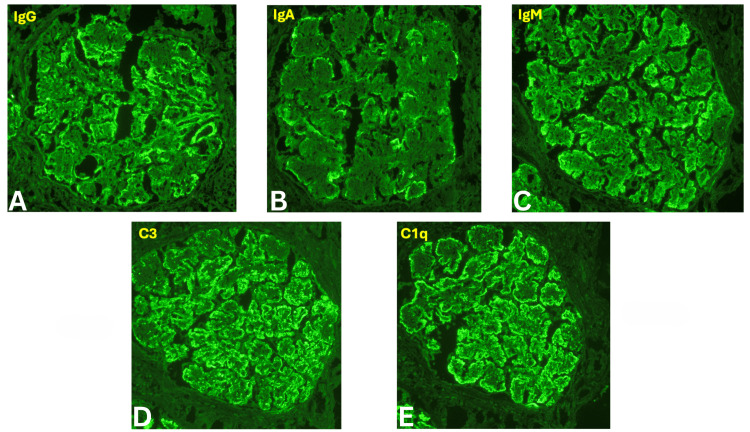
Immunofluorescence microscopy demonstrating IgG and IgM co-dominance (A) Immunofluorescence staining showing significant deposition of IgG. (B) Immunofluorescence staining showing weak deposition of IgA. (C) Immunofluorescence staining showing significant deposition of IgM. (D) Immunofluorescence staining showing significant deposition of C3. (E) Immunofluorescence staining showing significant deposition of C1q. IgG: immunoglobulin G, IgM: immunoglobulin M, IgA: immunoglobulin A Courtesy of Dr. Paisit Paueksakon and Dr. Tiffany L. Alley at the Vanderbilt University Department of Pathology, Microbiology, and Immunology

**Figure 7:**
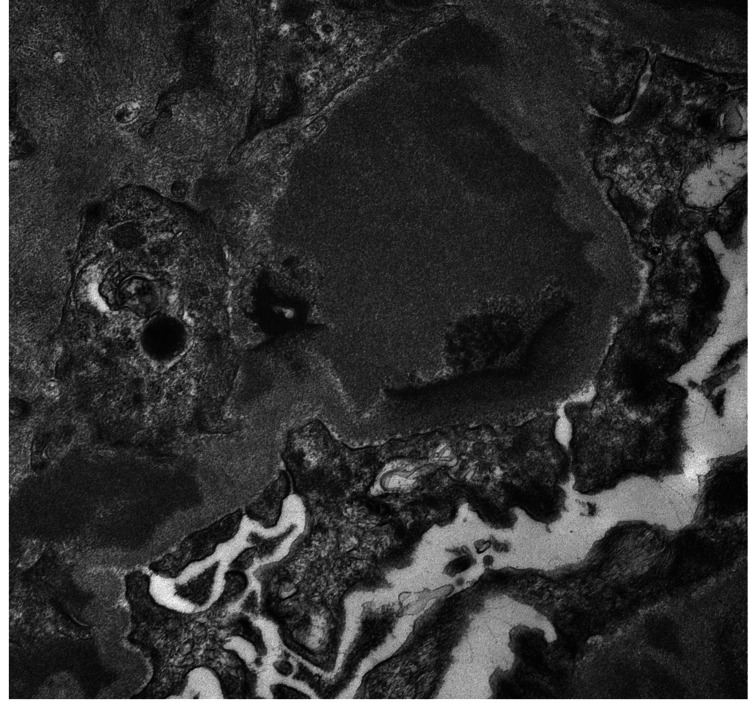
Subepithelial deposits seen on transmission electron microscopy Courtesy of Dr. Paisit Paueksakon and Dr. Tiffany L. Alley at the Vanderbilt University Department of Pathology, Microbiology, and Immunology

**Figure 8:**
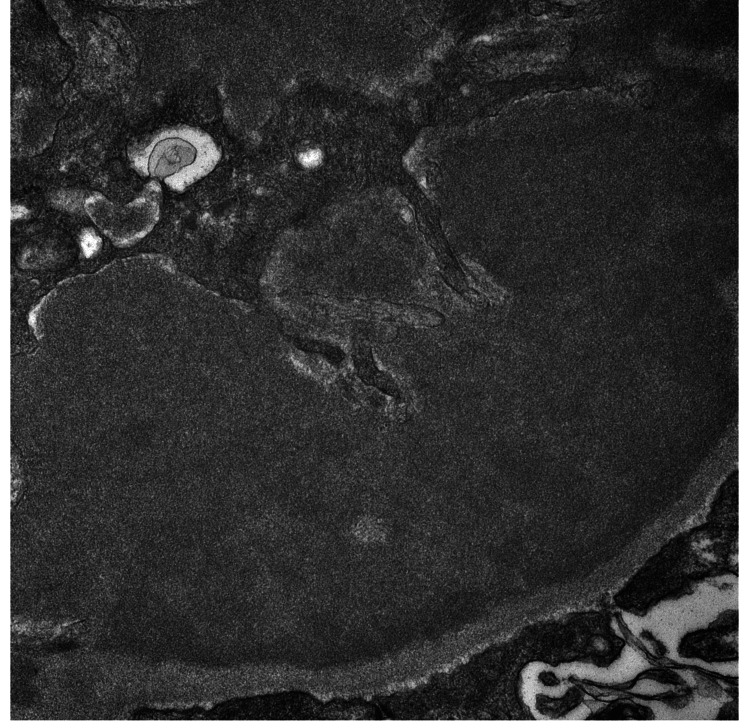
Subendothelial deposits with vague fibrillary and curvilinear substructures seen on transmission electron microscopy Courtesy of Dr. Paisit Paueksakon and Dr. Tiffany L. Alley at the Vanderbilt University Department of Pathology, Microbiology, and Immunology

A renal biopsy was performed, revealing 24 glomeruli, 10% to 20% interstitial fibrosis, and membranoproliferative glomerulonephritis with one fibrocellular crescent (Figure 2) and one cellular crescent (Figure 3). Additional findings included endocapillary hypercellularity (Figure 4), double contours, eosinophilic deposits (Figure 5), full-house staining with co-dominant IgG and IgM (Figure 6), and subepithelial and subendothelial deposits observed on transmission electron microscopy (Figures 7-8). Given the negative ANA, the findings are most consistent with type III polyclonal cryoglobulinemia.

The patient was started on pulse dose methylprednisolone for three days and transitioned to oral prednisone 60 mg once daily. Plasmapheresis was initiated every other day with fresh frozen plasma. Oncology was consulted for assistance with rituximab dosing and timing with plasmapheresis to avoid loss of rituximab during the exchange procedure. On the day of discharge, the patient was discharged on prednisone 20 mg daily, with expectant taper over the coming weeks. UPCR improved to 6.7 g from the previous 11.5 g with outpatient follow-up with nephrology and oncology. The patient ultimately underwent four weekly doses of rituximab 375 mg/m², followed by an additional two doses one and two months later. She now has a UPCR of 0.73 mg/dl with serum creatinine at 0.7 mg/dl.

## Discussion

MC in this patient is unusual as she had no obvious precipitating etiology, having denied any rash, skin ulcers, neuropathy, or worsening arthralgias. She also tested negative for hepatitis C virus (HCV) along with autoimmune diseases such as systemic lupus erythematosus, rheumatoid arthritis, and HIV. Additionally, hematologic abnormalities such as mild iron deficiency anemia and vitamin B12 deficiency were treated accordingly with no concerns for malignant etiologies. Therefore, this would be a case of idiopathic MC (IMC). This makes it a diagnostic challenge, as serologic testing for serum immunoglobulins is difficult, and IMC can also share morphology with other glomerular diseases [6-8].

It's important to note that the patient developed hydronephrosis during her pregnancy. According to the literature, there have been no known associations between hydronephrosis and glomerular diseases. Hydronephrosis is a normal occurrence during pregnancy and is less likely to cause any harm to the glomerulus. Additionally, other causes, such as alcoholic cirrhosis and polyclonal plasma cells (monoclonal gammopathy), have been identified as factors contributing to IMC [6,7]. In another case report, influenza vaccination was found to trigger IMC, but our patient did not have a history of receiving such vaccination [8]. The patient also showed no signs of infection, as blood and urine cultures were negative for seven days with no leukocytosis or fever, ruling out bacterial infections as a cause [9].

Our patient also tested seronegative for cryoglobulins along with being negative for hepatitis C. In one study, low cryoglobulin levels were often found to be polyclonal, unrelated to HCV infection, and recommended deeper investigation in the rheumatoid factor activity or signs of complement consumption [10]. Her rheumatoid factor was elevated, which can be seen in many conditions and could just be a sign of inflammation, especially with the anti-CCP antibodies being negative, thus ruling out rheumatoid arthritis. Another avenue to diagnose the rare causes of IMC could be testing for genetic and/or environmental factors [11,12]. Overall, this is an interesting case of IMC and poses the challenge of requiring further research into possible other causes of IMC.

## Conclusions

This case highlights the importance of considering IMC in patients with no risk factors. Our case is an atypical presentation, especially in the setting of an absence of skin manifestations. Further research is needed in areas of interest such as novel genetic markers, complement inhibitors, and immunomodulatory drugs to improve diagnostic and therapeutic challenges.
